# Psychometric performance of the PROMIS® depression item bank: a comparison of the 28- and 51-item versions using Rasch measurement theory

**DOI:** 10.1186/s41687-019-0131-4

**Published:** 2019-07-30

**Authors:** Sophie Cleanthous, Skye Pamela Barbic, Sarah Smith, Antoine Regnault

**Affiliations:** 1 Modus Outcomes Ltd, UK Office, Suite 210b, Spirella Building, Letchworth Garden City, SG6 4ET UK; 2 Faculty of Medicine, Department of Occupational Science and Occupational Therapy, University of British Columbia, Vancouver, BC Canada; 3 Department of Health Services Research & Policy, London School of Hygiene & Tropical Medicine, 15-17 Tavistock Place, London, WC1H9SH UK; 4 Modus Outcomes SAS FR Office, 61 Cours de la Liberte, 69003 Lyon, France

**Keywords:** PROMIS, Depression, Psychometrics, Rasch measurement theory, Rasch model

## Abstract

**Purpose:**

The aim of this study is to illustrate an example application of Rach Measurement Theory (RMT) in the evaluation of patient-reported outcome (PRO) measures. RMT diagnostic methods were applied to evaluate the PROMIS® Depression items as part of a series of papers applying different psychometric paradigms in parallel to the same data.

**Methods:**

RMT was used to examine scale-to-sample targeting, scale performance and sample measurement of two PROMIS depression item pools including respectively 28 and 51- items.

**Results:**

Sub-optimal but improved targeting was displayed in the 51-item pool which covered 27% of the range of depression measured in the sample compared to only 15% in the 28-item bank, further reducing the sample percentage with lower depression not covered by the scale (28% Vs 34%). Satisfactory scale performance was observed by the 28-item bank with marginal item misfit. However, deviations from the RMT criteria in the 51-itempool were observed including: 9 reversed thresholds; 12 misfitting items and 12 item-pairs displaying dependency. Overall reliability was good for sets of items (Person Separation Index = 0.93 and 0.95), but sub-optimal sample measurement (17% Vs 19% fit residuals outside of the recommended range).

**Conclusions:**

The RMT approach in this exercise provided evidence that compared to the 28-item bank, the extended 51-item version of the PROMIS depression, improved sample-to-scale targeting. However, targeting in the lower end of the concept of interest remained sub-optimal and scale performance deteriorated. There may be a need to improve the conceptual breadth of the construct under investigation to ensure the inclusion of items that capture the full range of the concept of interest for this context of use.

## Background

The rising profile of including the patient perspective in clinical outcome assessment has consequently increased interest in patient reported outcome (PRO) instruments and techniques of evaluating their scientific rigor [1, 2]. Scores generated by PRO instruments are increasingly used as central outcome variables upon which important decisions are made related to patient care. Therefore, it is essential to assess whether they are fit for purpose as failure to do so could potentially lead to incorrect interpretations being drawn about patient care [1, 3]. A fundamental step in PRO evaluation is to examine whether an instrument comprehensively captures the concept of interest in the intended context of use [4]. Additionally, it is essential to assess whether summing individual items is “psychometrically sound” and whether—and to what extent—generated scores satisfy a priori reliability and validity criteria [5–7].

Different psychometric paradigms are available for developing and evaluating the scientific rigour of PRO instruments [1]. These include traditional psychometrics based on the theoretical Classical Test Theory (CTT) [8, 9] and more recently modern psychometric paradigms offering mathematically testable models: the Rasch Measurement Theory (RMT) [10, 11] and Item Response Theory (IRT) [12, 13]. CTT is grounded in Steven’s definition of measurement as “the assignment of numerals to objects or events according to some rule,” which proposes that in measurement a person’s observed score is the sum of their true and error estimates yet where the true score is theoretical [9]. Traditional psychometric analysis examines raw scores without weighing or standardization against theoretical measurement [8]. Modern psychometric paradigms on the other hand are grounded in Thurstone’s measurement criteria which include interval scaling and measurement invariance [14, 15] and offer mathematical testable logistic models against which measurement properties of rating scales can be examined [1].

This study constitutes part of a parallel exercise coordinated by the International Society for Quality of Life Research ISOQOL’s Psychometrics Special Interest Group (SIG) aiming to demonstrate the potential of different psychometric methodologies (CTT, IRT and RMT) for developing and evaluating PROs using the same exemplar instrument. The papers describing the three parallel studies were reviewed by members of the Psychometrics SIG and the ISOQOL board. The Patient-Reported Outcomes Measurement Information System (PROMIS) calibrated depression item bank [16, 17] was the chosen PRO example for this parallel exercise. The PROMIS depression item bank consists of a 28-item version calibrated from a larger pool of 56 well-performing items. PROMIS item calibration was completed by applying IRT models and CTT techniques to an initial 518-item bank collated from 78 depression scales [17]. Items for the depression bank were generated through an iterative process involving literature searches, conceptual framework development, expert review, focus groups and cognitive debriefing [17]. As the PROMIS depression authors suggested, validation of item banks should be an on-going process [17].

In this study we present an evaluation of the PROMIS depression item bank using RMT methods [3, 10]. Similar to the IRT approach used in the development and calibration of the PROMIS item banks [18] RMT offers a mathematical model that defines how a set of items perform to generate reliable and valid measurements [10]. The Rasch model postulates that the probability of response to an item is always a function of the difference between an item’s difficulty and a person’s ability [3, 10, 11]. The Rasch model defines how a set of items should perform to generate reliable and valid measurements, and RMT analysis examines the extent to which the observed scores data “fit” the scores expected by the Rasch model [3].

Both IRT and RMT are used to generate data focusing on item responses within scales. Despite the similarities of these two new modern psychometric approaches, they are characterized by a fundamental difference that is key in scale evaluation and modification [1]. IRT is a statistical modeling paradigm that aims to find the best measurement model to fit the observed data. RMT on the other hand is a diagnostic paradigm that aims to assess the extent to which a set of items both conform to the requirement of the Rasch model, based on subjects’ responses, and identify potential anomalies that do not fit the RMT expectations [1, 19]. In practice, anomalies within the RMT paradigm are resolved by revisiting the item content and appraising the qualitative and quantitative evidence to further understand the disparity between expected and observed scores of an evaluated scale [3]. Evaluating the PROMIS depression items using RMT methods can therefore provide an additional perspective to the on-going validation of the PROMIS depression item bank for application in research, clinical practice, and policy.

## Methods

### Sample

The analyses described here are secondary analysis of data initially collected in the PROMIS Wave 1 cohort [16, 17]. Recruitment took place at four PROMIS sites (University of Pittsburgh, University of North Carolina-Chapel Hill, Stanford University, and Duke University) and online via YouGovPolimetrix.com—a non-partisan online polling firm administering surveys for market research and political polling. As PROMIS item banks are targeted for use in clinical research (including clinical trials, observational research, and epidemiological studies), authors reported assembling a representative sample with a diverse severity of emotional distress.

### PROMIS depression item Bank

The development of the PROMIS depression item bank has been described in detail elsewhere [17]. Items are rated on a 5-point frequency scale (never, rarely, sometimes, often, always) within a seven-day recall period with a model-based scoring system with higher values reflecting greater depression [17]. The 28-item bank comprised a single factor combining items from cognitive (*n* = 17), affective (*n* = 9), behavioral (n = 1) and suicidal (n = 1) conceptual domains. The current analyses use data from the extended 56-item PROMIS depression item bank. Five items were eliminated for intellectual property issues, leaving 51 items in the pool available for analysis. As an item bank relates to items that have been calibrated, the 51-item set will be referred to as an item pool.

### Rasch measurement theory (RMT) analysis

The original simple logistic Rasch model [11] postulates that the probability of a positive response to a dichotomous (yes/no) item is a logistic function of the relative difference between the respondent (person) location and the item location on the measurement continuum. The Rasch model—which articulates how measurements of constructs can be derived from responses to items—postulates that the odds of a “yes” response correspond to the probability of a “yes” response divided by the probability of a “no” response. This approach outlines a natural logarithm, where the person and item locations are additive in log-odd units (logits), thus transforming scores into an interval scale. [3] The dichotomous model was subsequently expanded to polytomous data which conceptually and mathematically reflect an extension of dichotomous data [3]. Andrich [10] subsequently developed the rating scale Rasch model, the unrestricted version of which was used by this study [10].

RMT analysis uses the Rasch Model as the criterion against which scale performance is evaluated [1, 3, 10]. Effectively, RMT analysis examines the extent to which observed raw scores (responses to scale items) satisfy the a priori criteria and match the scores expected by the Rasch model. The evaluation of a rating scale using RMT analysis has been used commonly to develop and test measures in health, including psychiatry [20] using three broad aims: the evaluation of the scale-to-sample targeting, scale performance, and sample measurement. The tests and information collected to address these aims have been described in detail elsewhere [3] and in brief below.

### Scale-to-sample targeting

Scale-to-sample targeting refers to the extent to which a scale’s items are able to measure the sample’s ability range (in this case depression). Targeting was evaluated through examination of the relative person and item distributions on the same continuum [21, 22] both graphically and numerically. RMT analysis orders items and responders in hierarchical order according to their relative difficulty (item locations) and relative ability (person location) on the same interval continuum of logits. The relative distributions inform the adequacy of the sample for evaluating the scale and the adequacy of the scale for measuring the sample, the better the ranges are matches for each other, the greater the potential for precise person measurement.

### Scale performance

Five components of scale performance were examined [23].

#### Do the response categories work as intended?

Each PROMIS item is scored on a 5-point frequency response scale as follows: “1 = never”; “2 = rarely”; “3 = sometimes”; “4 = often”; “5 = always.” Respondents with higher levels of the construct (i.e., higher depression) are expected to endorse the higher response categories, while respondents with lower levels of the construct (i.e., lower depression) are expected to endorse the lower response categories. Thresholds represent the point between two response categories (i.e., the place where a person is equally likely to endorse either of the two response categories). Threshold ordering is expected to reflect the intended category ordering. Disordered thresholds signify response categories may not be working as intended. This may in turn affect scale score interpretations and scale validity, where higher scores may not necessarily reflect higher levels of the concept of interest [24].

#### Do the items map out a continuum of depression?

An optimal scale is expected to comprise of a continuum representing the construct under measurement (e.g., depression) with marked components articulated as items located at different points on the continuum [25–27]. The different locations are expected to cover the entire range of the construct range (i.e. scale items are intended to measure and equally representing all levels of within a construct) [23, 28]. Therefore, item location, spread, proximity to each other and precision are examined in RMT analysis, to determine the extent to which the set of items map out a continuum for measurement from less to more ability [21, 23].

#### Do the items define a single construct?

Within the RMT analysis, item responses are examined to assess the cohesiveness of the measurement continuum and therefore the legitimacy of the scale [23, 28]. Fit statistics identify the extent to which items work well together to define a single variable. Item fit statistics summarize the differences between observed scores and expected responses for individuals (i.e. the item-person interaction [fit residual]) and different ability groups (i.e. the item-construct interaction [chi square values]) [3]. Criteria suggest fit residuals should lie within the recommended range of − 2.5 and + 2.5, and Chi square values should be non-significant [3, 21]. Item characteristic curves (ICC) are graphical indicators of fit which are used to complement the interpretation of the fit residuals and chi square probabilities [23, 29].

#### Do responses to one item bias responses to other items?

The RMT expects item independency such that the response to one item should not influence or determine the response to another. High residual (i.e. observed - expected = residual) correlations highlight “local dependency” or item response bias which can artificially inflate reliability [21, 22]. Response bias was assessed in line with the r > 0.30 rule of thumb, which indicates a > 9% shared variance between a pair of items, suggesting local dependence [30].

#### Are the scale items stable between different groups?

The extent to which items are stable across different sample subgroups was assessed through differential item functioning (DIF) [3, 23, 31]. An item shows differential functioning if the expected response for two respondents who have the same level on the measured construct but from two different groups (age, sex) differs. DIF was investigated by examining the observed response differences between class intervals within groups. DIF was examined using analysis of variance (ANOVA), assessing item scores between the sample groups and across the different class-intervals where a significant *p*-value for differences between subgroups is taken to indicate DIF [3, 24]. The performance of the items was tested for stability across gender (male/female) six age groups (18–30, 31–40, 41–50, 51–60, 61–70 and 70+); race (white/non-white) and Hispanic ethnicity (Hispanic/non-Hispanic).

### Sample measurement

Two components of person measurement were examined: the Person Separation Index (PSI), and the extent to which individuals’ responses were consistent with expectation. The PSI, which indicates a scale’s ability to detect differences in the levels of the construct within the sample, is a numerical indicator computed as the ratio of the variation of person estimates relative to the estimated error for each person [31]. PSI scores range between 0 and 1, where a 0 score indicated all error and a 1 score no error [3].

The extent to which individuals’ responses met the expectations of the Rasch model was examined with fit statistics. Person fit residuals summarize the difference between observed scores and expected responses for each person i.e., the person-construct interaction [3, 23]. Person fit residuals were examined with reference to the “rule of thumb,” expecting 99% of the sample to produce a fit residual between − 2.5 to 2.5. Fit residuals outside this range indicate potential problematic measurements for those persons [3, 23].

### Analysis procedure

Data were analyzed in using RUMM2030 [11], one of several available software programs that provides graphical and statistical item analyses using Rasch unidimensional models for measurement. In line with the methods described above, the initial RMT analysis of the 28-item bank identified limited measurement and precision on the floor of the scale. We therefore hypothesized that adding new items could improve measurement performance by expanding the range of depression measured by the PROMIS items. For this, the extended item pool of 51 items* (i.e., original 28 + 23 items) was analyzed with RMT and performance compared to original 28-item bank. For brevity, results are presented in two stages: in stage 1, the extent to which the 51-items satisfy the RMT criteria, and in stage 2, the relative measurement performance of the 51-item and the 28-item pools. A summed total score was calculated for both the 28 and 51-item pools. Five items were eliminated from the 56-item bank due to potential intellectual property issues.

## Results

### Sample

In the sample, described elsewhere [16, 17], 8% of participants were of a clinical population and 92% of a general population. Secondary analysis was performed on a subsample (*n* = 825) of patients ranging in age 18–88 years (mean = 50.91, SD = 18.92). Of these 51% were female, and 79% white, 10% Hispanic. The sample size reported between the two different stages of analyses (both on scale and on item level) varies slightly, as RMT analyses estimates are calculated on the basis of available data and excluding extreme scores located at the floor and ceiling.

### Stage 1 RMT analysis: examination of the PROMIS 51-item pool measurement performance

#### Scale-to-sample targeting

**Fig. 1:**
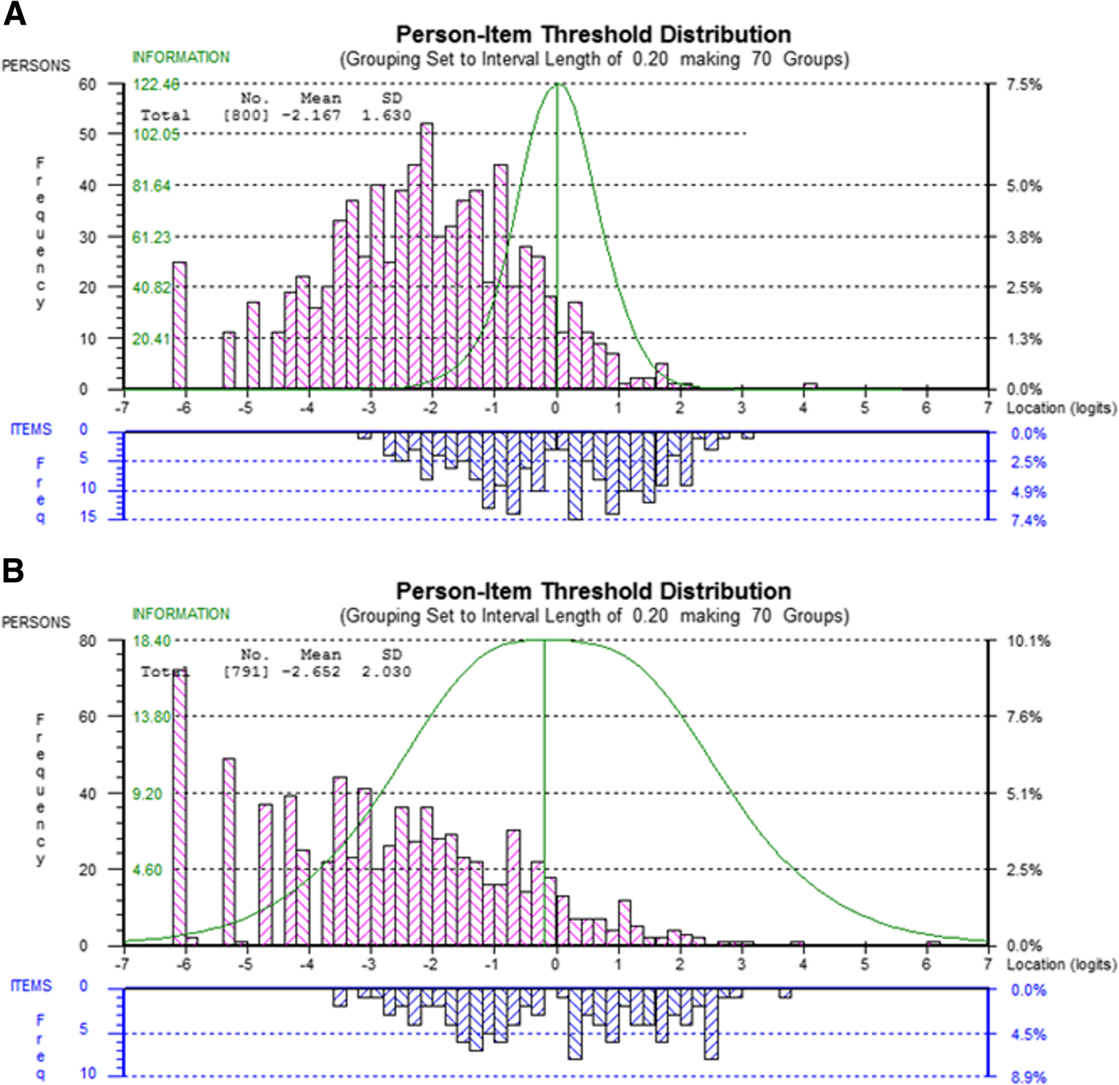
Scale-sample targeting: top histograms (pink bars): sample’s distribution of depression measurements (person locations) measured by the PROMIS 51 items in (**a**) and the PROMIS 28 in (**b**). Person measurements reflecting lower ability (lower depression) are represented by histogram bars on the left and person measurement reflecting higher ability (higher depression) by the histogram bars on the right. Lower histograms (blue bars): scale distribution of item threshold location estimates for the 51 items in (**a**) and the 28 items in (**b**). Histogram bars on the left represent the easiest items (requiring lower levels of depression to be positively endorsed) and histogram bars on the right represent the more difficult items (requiring higher levels of depression to be positively endorsed). Green curve: represent an inverse function of the standard error associated with measurement, it reflects scale’s precision and shows the location on the continuum the scale performs at its best, indicating that the location of item thresholds and persons is well matched

Person locations ranged approximately between − 6 to 4 logits relative to the item threshold locations, ranging approximately between − 3 to 3 logits, and covering 60% of the depression measured in the sample (Table 1). Graphical review of targeting **(**Fig. 1a, b) indicated item bunching and sub-optimal measurement for the persons with lower depression scores. Sample measurements (*n* = 224, 28%) located below − 3.13 logit on the left of the continuum had no matching items.

**Table 1:** Targeting, reliability and sample measurement validity

	PROMIS-51	PROMIS-28
Targeting		
Range (mean): person measurements	−6.14 to 4.20 (−2.17)	− 6.00 to 6.03 (2.65)
Range (mean): item locations	−1.58 to 1.18 (0.00)	−0.68 to 1.16 (0.00)
Range (mean): item thresholds	−3.13 to 3.12 (0.00)	− 3.46 to 3.75 (0.00)
Item location/sample coverage	27%	15%
Item threshold/sample coverage	60%	60%
Floor/ceiling effects %	3/0%	7/0%
Reliability		
Person Separation Index (PSI)	0.95	0.93
Sample Measurement Validity		
Range (mean): person fit residual	−7.49 to 5.76 (−0.32)	−6.48 to 4.99 (− 0.40)
Measurements outside recommended range	*n* = 139 (17%)	*n* = 148 (19%)
Item-trait interaction: Chi-square (p-value)	1720.3 (*p* < 0.001)	401.5 (p < 0.001)

### Scale performance

#### Do the response categories work as intended?

Nine items displayed disordered response thresholds (Table 2), with the “rarely” response category most consistently not working as intended.

**Table 2:** PROMIS 51 Thresholds ordering, item location estimates, item fit statistics and item dependency (items ordered by location)

Item label		Disordered Threshold^a^	Location^b^		Fit Statistics		Res. r^e^
			Estimate	SE	Fit Residual	Chi-Square	
15	I disliked the way my body looked		−1.58	0.04	12.21	261.75	≤0.30
24	I felt like being alone		−1.04	0.05	4.92	36.02	≤0.30
3	I felt that I had no energy		−0.96	0.05	3.32	15.34	0.40
18	I got tired more easily than usual		−0.75	0.05	3.40	13.82	0.35
26	I felt disappointed in myself		−0.54	0.05	−2.54	13.43	≤0.30
43	I felt slowed down		−0.54	0.05	1.11	18.93	0.35
31	I felt discouraged about the future		−0.50	0.05	−0.64	10.97	≤0.30
36	I felt unhappy		−0.49	0.05	−6.49	38.30	0.33
11	I ate more than usual		−0.45	0.05	12.59	179.81	≤0.30
46	I felt pessimistic		−0.45	0.05	−0.76	5.33	≤0.30
17	I felt sad		−0.43	0.05	−3.69	32.23	0.33
12	I had mood swings		−0.42	0.05	0.28	9.23	≤0.30
8	I felt that everything was an effort		−0.39	0.05	1.73	12.67	≤0.30
54	I felt emotionally exhausted		−0.39	0.05	−4.46	25.83	≤0.30
23	I had trouble feeling close to people		−0.35	0.05	−0.34	8.91	≤0.30
28	I felt lonely		−0.34	0.05	0.19	6.82	0.33
56	I had trouble enjoying the things that I used to enjoy		−0.23	0.05	−2.88	12.27	≤0.30
47	I had trouble keeping my mind on what I was doing		−0.21	0.05	−0.58	11.59	0.36
29	I felt depressed		−0.15	0.05	−6.05	38.93	≤0.30
35	I found that things in my life were overwhelming		−0.13	0.05	−3.29	21.52	≤0.30
7	I withdrew from other people		−0.12	0.05	−0.74	12.98	≤0.30
42	I felt ignored by people		−0.07	0.05	−0.20	10.77	≤0.30
30	I had trouble making decisions		−0.01	0.06	−2.58	25.81	≤0.30
21	I felt that I was to blame for things		0.00	0.06	−2.71	20.76	≤0.30
48	I felt that my life was empty	X	0.00	0.05	−2.31	7.04	≤0.30
50	I felt guilty		0.01	0.05	−0.60	7.11	≤0.30
22	I felt like a failure	X	0.03	0.05	−4.18	24.61	≤0.30
14	I felt that I was not as good as other people		0.04	0.05	−1.12	7.28	≤0.30
16	I felt like crying		0.10	0.06	0.10	5.01	0.44
20	My thinking was slower than usual		0.12	0.06	−0.17	19.5	0.40
38	I felt unloved		0.14	0.06	0.31	8.43	≤0.30
27	I felt that I was not needed		0.17	0.06	−1.63	11.6	≤0.30
1	I reacted slowly to things that were done or said		0.19	0.06	1.22	12.15	≤0.30
52	I had trouble thinking clearly		0.19	0.06	−1.16	11.37	0.40
5	I felt that I had nothing to look forward to		0.27	0.06	−3.69	14.35	≤0.30
55	I felt I needed help for depression	X	0.28	0.06	−2.64	12.98	≤0.30
53	I had little desire to eat		0.30	0.06	5.85	64.88	≤0.30
45	I felt that nothing was interesting		0.31	0.06	−2.54	14.44	≤0.30
4	I felt worthless		0.37	0.06	−4.02	35.41	≤0.30
37	I was unable to do many of my usual activities		0.39	0.06	3.37	18.07	≤0.30
49	I lost weight without trying		0.39	0.06	9.68	408.14	≤0.30
6	I felt helpless		0.40	0.06	−4.27	35.22	0.33
41	I felt hopeless		0.45	0.06	−5.71	41.52	0.33
9	I felt that nothing could cheer me up		0.46	0.06	−5.00	33.75	≤0.30
19	I felt that I wanted to give up on everything	X	0.49	0.06	−2.69	16.56	≤0.30
44	I felt upset for no reason		0.63	0.06	−3.47	14.37	≤0.30
34	I had crying spells	X	0.84	0.07	2.03	8.78	0.44
32	I wished I were dead and away from it all	X	0.89	0.07	−2.08	7.1	0.53
39	I felt I had no reason for living	X	0.93	0.07	−2.33	11.11	0.53
33	I thought about suicide	X	0.96	0.08	−0.10	9.98	0.47
40	I felt that others would be better off if I were dead	X	1.18	0.07	0.30	15.58	≤0.30

^a^X: disordered thresholds; ^b^item location estimates and standard error (SE); ^c^Fit residuals, numbers outside recommended range of −2.5 to + 2.5 are printed in bold; ^d^chi-square value; when statistically significant at ≤0.01 after Bonferroni correction number is printed in bold; ^e^maximum Residual correlation (Res. r) for each item when value is > 0.30 rule of thumb the actual number is reported

#### Do the items map out a depression ability continuum?

**Fig. 2:**
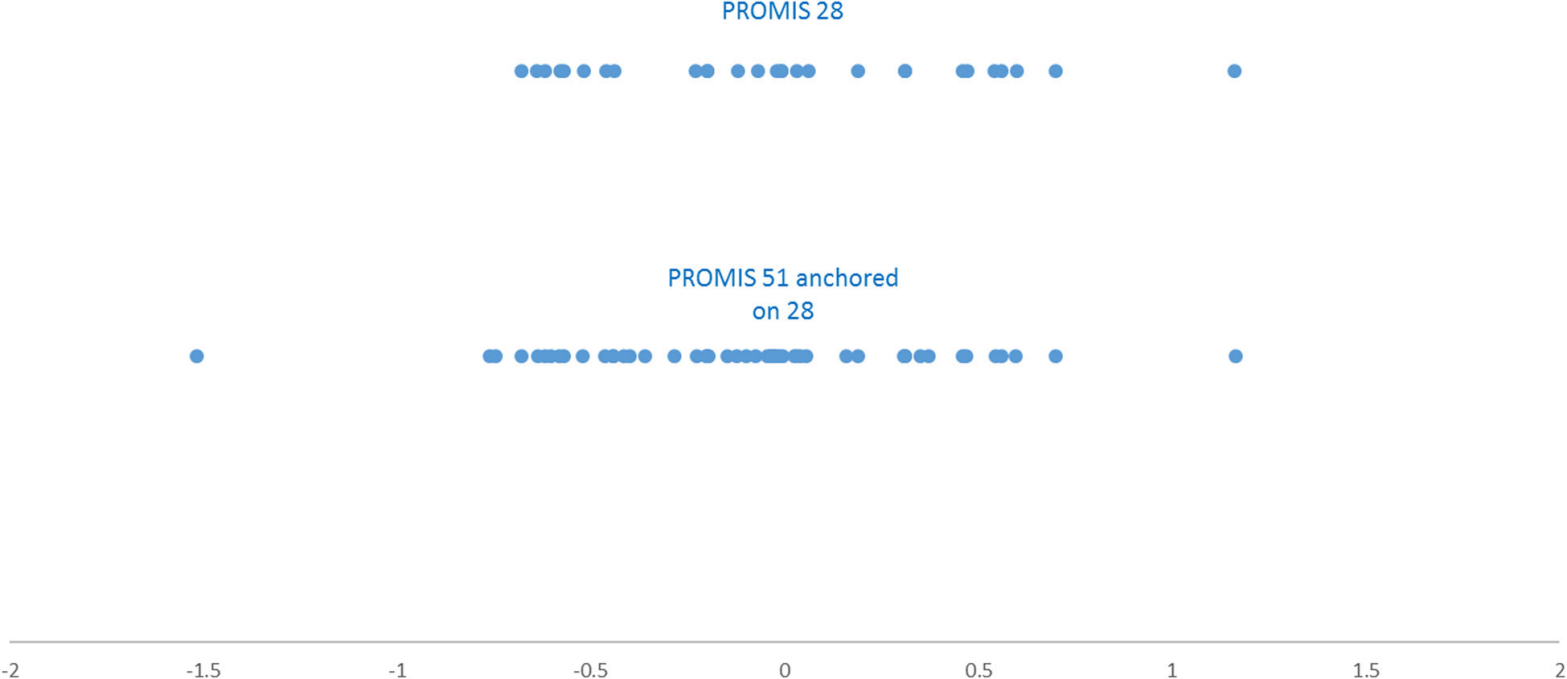
Plot of relative items location of the PROMIS 51 and 28 item pools. The blue dots represent the item locations for each item within the PROMIS 28 and PROMIS 51 (as anchored on the PROMIS-28 measurement continuum) item banks. The items located on the left of the continuum are the easiest (i.e. are relevant to persons with lower levels of depression); items towards the right are the most difficult (i.e. are relevant to person with higher levels of depression). The PROMIS 51 items cover parts of the continuum (<− 0.75) and around (− 0.50 to 0.00) where the PROMIS 28 item distribution displays item gaps, as well as smaller gaps of the 28-item pool along the continuum

Item locations for the PROMIS-51 continuum ranged between 1.58 to 1.18 logits **(**Table 1**)** and showed some overlap. For example, five items (items: 21, 30, 42, 48 and 50) were situated on within less 0.08 logits of each other and three items (items: 6, 9, and 41) within 0.06 logits of each other (Table 2, Figure 2).

#### Do the items define a single construct?

**Fig. 3:**
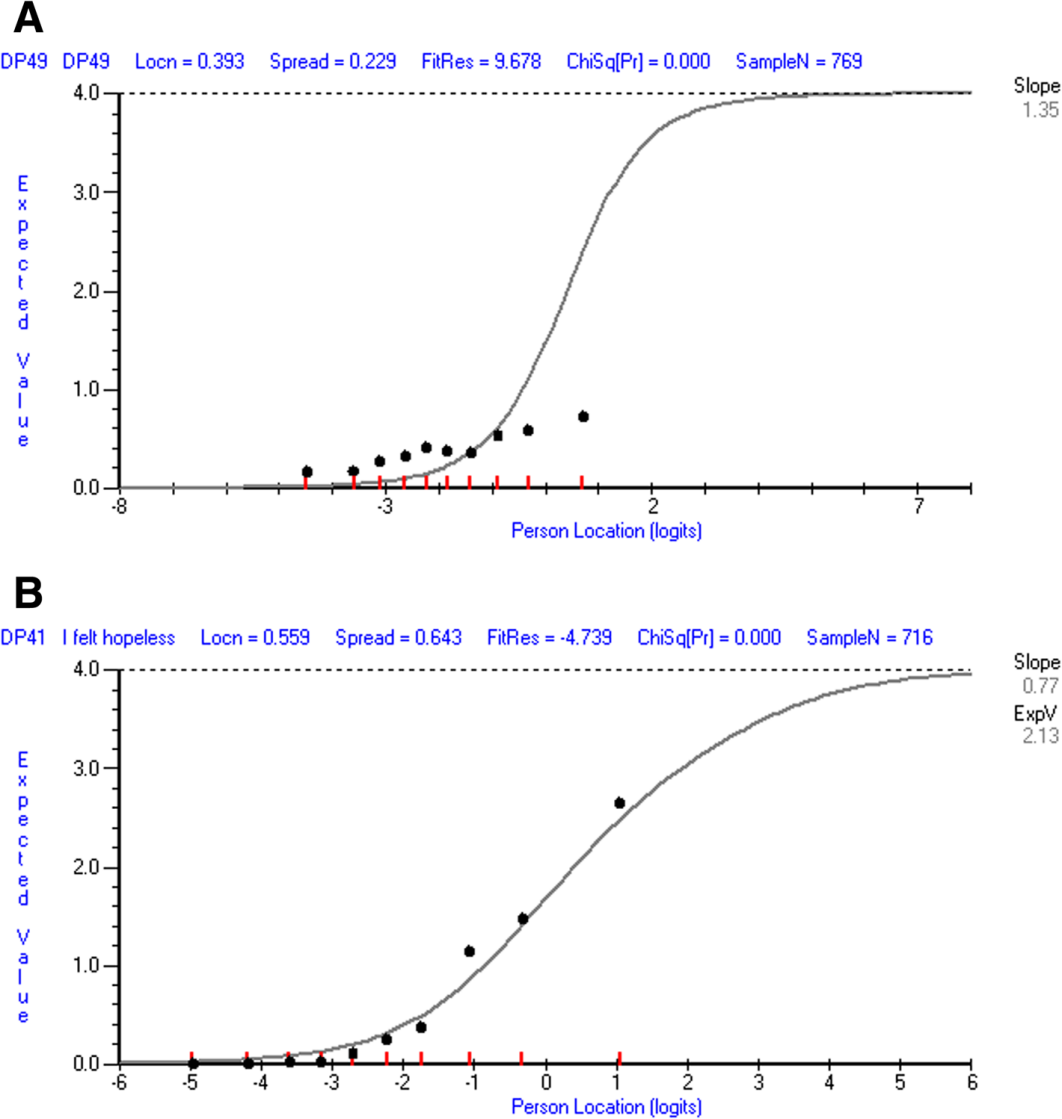
Exemplar Item Characteristic Curves (ICC) for items displaying the worst Statistical Misfit. Item characteristic curves for the items displaying the worst statistical misfit. The y-axis represents the scores expected by the Rasch model and the x-axis the depression measurement continuum of the sample. The ICC plots the observed scores in each of the 10 class intervals of depression (the black dots) and the scores expected by the Rasch model at each level of the measurement continuum (the curve). (**a**) item 49 “I lost weight without trying” of the 51-item bank indicating evident underestimation of depression as observed scores at higher person location logits are located below the expected curve and scores at lower person location logits higher than the expected curve. (**b**) item 41 “I felt hopeless” of the 28-item bank indicating marginal overestimation of depression as observed scores at higher person location logits are located above the expected curve and observed scores

Twenty items displayed fit residual outside the recommended range, twelve of which also displayed significant chi square values (Table 2). Graphical review of the ICCs indicated overestimation of depression for items 17, 29 and 36 and underestimation of depression (i.e. observed scores were lower than expected at higher depression-ability locations and higher than expected at lower depression-ability locations) for items 11, 15, 24, 49 and 53. Exemplar ICCs are displayed in Fig. 3**.**

#### Do responses to one item bias responses to other items?

Twelve item pairs produced residual correlations above the recommended range, suggesting possible response bias between them. The highest residual correlation (r = 0.53) identified was between items 32 and 39 (Table 2).

#### Are the scale items stable between different groups?

No item displayed DIF by race or Hispanic ethnicity, whereas two items (items: 15 and 16) displayed DIF by gender, and ten items (items: 11, 12, 18, 20, 35, 37, 43, 47, 54 and 56) displayed DIF by age group (*p* < 0.01).

### Sample measurement

The PROMIS-51 displayed high reliability and sample separation (PSI = 0.95) and suboptimal sample measurement as a total of 139 (17%) individuals in the sample fell outside the recommended range.

### Stage 2 RMT analysis: comparative measurement performance of the 51 and 28-item pools

#### Scale-to-sample targeting

Even though the 51-item pool did not resolve targeting issues of the PROMIS 28-item pool, it did provide relative improvements to the measurement of people on the floor of the scale. Graphical review suggests the relative limitation of the 28-item pool (Fig. 1b) to measure depression for people with lower depression scores relative to the 51-item pool (Fig. 1a). In line with this, the sample mean is further skewed away from the item mean, floor effects rise to 7 from 3% in the 28-item bank **(**Table 1**)** as do the sample measurements outside the scale range which amount to 34% (*n* = 272) in the 28-item pool as compared to 28% (*n* = 224) 51-item pool. Figure 2 demonstrates how additional items improved to targeting as they expanded item coverage on the left of the continuum and filled in some of the gaps on the PROMIS 28-item pool measurement continuum. This improvement is further demonstrated numerically: the relative percentage coverage of the sample measurement by item location was 15%, in comparison to 27% in the 51-item pool (Table 1).

### Scale performance

As compared to the 51, the 28-item pool satisfied more criteria of RMT in relation to scale performance.

#### Do the response categories work as intended?

Thresholds for only one item (compared to nine in the 51-item version) were not consistently ordered sequentially, suggesting the “rarely” response category was problematic for this item (Table 3).

**Table 3:** PROMIS 28 Thresholds ordering, item location estimates, item fit statistics and item dependency

Item label		Disordered Thresholds^a^	Location^b^		Fit Statistics		Res. r^e^
			Estimate	SE	Fit Residual	Chi-Square	
26	I felt disappointed in myself		−0.68	0.06	−0.18	16.89	≤0.30
31	I felt discouraged about the future		−0.64	0.06	1.36	11.02	≤0.30
36	I felt unhappy		−0.62	0.06	−4.86	26.40	≤0.30
46	I felt pessimistic		−0.58	0.06	2.15	11.32	≤0.30
17	I felt sad		−0.57	0.06	−1.05	15.03	≤0.30
54	I felt emotionally exhausted		−0.52	0.06	−0.2	6.74	≤0.30
23	I had trouble feeling close to people		−0.46	0.06	1.86	9.27	≤0.30
28	I felt lonely		−0.44	0.06	2.36	9.85	≤0.30
29	I felt depressed		−0.23	0.06	−3.63	16.81	≤0.30
7	I withdrew from other people		−0.20	0.06	2.39	13.99	≤0.30
35	I found that things in my life		−0.20	0.06	0.85	6.40	≤0.30
42	I felt ignored by people		−0.12	0.06	2.44	15.17	≤0.30
30	I had trouble with decisions		−0.07	0.06	1.81	15.16	≤0.30
50	I felt guilty		−0.02	0.06	2.17	22.41	≤0.30
21	I felt that I was to blame for		−0.01	0.06	−0.19	10.5	≤0.30
48	I felt that my life was empty	X	−0.01	0.06	−0.59	15.51	≤0.30
14	I felt that I was not as good as other people		0.03	0.06	1.08	4.60	≤0.30
22	I felt like a failure		0.06	0.06	−3.7	16.23	≤0.30
27	I felt that I was not needed		0.19	0.06	−0.26	5.08	≤0.30
5	I felt that I had nothing to look forward to		0.31	0.06	−1.51	12.54	≤0.30
45	I felt that nothing was interesting		0.31	0.06	1.21	5.06	≤0.30
4	I felt worthless		0.46	0.07	−2.44	22.97	≤0.30
6	I felt helpless		0.47	0.06	−1.63	20.05	≤0.30
9	I felt that nothing could cheer me up		0.54	0.07	−3.47	21.44	≤0.30
41	I felt hopeless		0.56	0.07	−4.74	35.32	≤0.30
19	I felt that I wanted to give up on everything		0.60	0.07	−1.05	8.23	≤0.30
44	I felt upset for no reason		0.70	0.07	−0.29	12.75	≤0.30
39	I felt I had no reason for living		1.16	0.08	−1.29	14.79	≤0.30

^a^√: ordered thresholds; X: disordered thresholds; ^b^item location estimates and standard error (SE); ^c^Fit residuals, numbers outside recommended range of −2.5 to + 2.5 are printed in bold; ^d^chi-square value; when statistically significant at ≤0.01 after Bonferroni correction number is printed in bold; ^e^maximum residual correlation (Res. r) for each item when value is > 0.30 rule of thumb the actual number is reported

#### Do the items map out a depression ability continuum?

The item location range of the 28-item pool—ranging from − 0.68 to 1.16 logits—was approximately 1 logit narrower than the 51-item pool ranging from − 1.58 to 1.18 logits (Table 1). Item locations consistently indicated some overlap: for example, five items (items: 14, 21, 22, 48 and 50) were situated within 0.08 logits and three items (items: 9, 19 and 41) within less than 0.05 logits (Table 3).

#### Do the items define a single construct?

Fewer items displayed misfit in the 28-item pool, as five items displayed fit residuals outside the recommended range, only one of which also displayed a significant chi square value (Table 3). The item characteristic curve for this item (Fig. 3b) indicated overestimation of depression: observed scores were higher than expected at higher depression-ability locations and lower than expected at lower depression-ability locations.

#### Do responses to one item bias responses to other items?

In comparison to the longer item pool, no item bias was identified in the 28-item pool as no items pairs produced residual correlations above 0.30 (Table 3).

#### Are the scale items stable between different groups?

Compared to the longer item pool, the 28-item displayed minimal DIF as no items displayed DIF by gender, race or Hispanic ethnicity and only one item (item 35) displayed DIF by age group (*p* < 0.01).

### Sample measurement

Sample measurement was similar in the two PROMIS item-pools. The PSI of the 28-item pool was marginally lower (PSI = 0.93) than that of the 51-item and the validity of sample measurement marginally worse as the percentage of person fit residuals outside the recommended range rose to 19% (Table 1).

## Discussion

This study was part of a parallel exercise aiming to compare three different psychometric paradigms (CTT, IRT, RMT) and explore the psychometric properties of the PROMIS depression item bank [17] using RMT [3.10]. RMT analysis focuses on the examination scale-to-sample targeting; scale performance and sample measurement exploring the disparities between the observed scores and those expected by the RMT model. [3] Using this approach, improvements were observed with the 51-item version of the PROMIS depression inventory, compared to the original 28-item version. However, RMT also revealed shortcomings of the extended item set, specifically suboptimal sample-to-scale targeting.

Specifically, RMT evaluation of the PROMIS depression 28-item bank indicated overall adequate scale performance, but importantly revealed suboptimal targeting. More than a third of the sample was located at the lower end of the depression continuum where few items were identified to capture the individuals in this range. In other words, the 28-item bank is not well targeted for those with lower depression for whom interpretations using the PROMIS depression would be associated with limited precision (i.e., higher standard error). This was further supported by the high percentage of person fit residuals identified outside the recommended range, indicating problematic measurement for nearly 20% of the sample.

The extended 51-item PROMIS depression item pool was also analyzed to examine whether an additional 23 items improved the sample-to-scale targeting by extending the item continuum range. Although the 51-item pool showed improved targeting, problems were not resolved. In fact, the additional items impacted negatively on the measurement properties of the item bank. Specifically, the extended item pool appeared less statistically cohesive, suggesting the set of items were not measuring a single construct. As well, evidence suggested item dependency and problems with the ordering of item response thresholds.

Despite the short-comings revealed, the approach suggests possible ways to remedy the existing scale. Exploration of the construct at the lower end of the continuum and the identification of items to capture the concept of interest for the population under investigation may optimize the scale for the intended use. We hypothesize that a conceptually driven effort to fill in the item gaps will improve the content validity of both the PROMIS depression 28-item and 51-item pools [1, 6]. Review of the item development process indicates that initial pool of items was inductively categorized into conceptual domains (including 46 depression facets) before undergoing further standardization and calibration. [17] However, as Pilkonis et al. (2011) note, certain trade-offs were necessary to reach the assumption of unidimensionality and a single factor scale—a requirement of IRT models used to calibrate and evaluate these items. [17] Subsequent focus groups conducted with major depression patients [16] supported the multidimensional domains which further suggested additional conceptual categories of depression.

In addition to the issue of multiple conceptual domain, the diversity of items within the PROMIS depression item bank further fails to match the diversity of concepts within the conceptual framework [17]. As reported by Pilkonis et al. (2011), only the proportion of affective items was consistent, whereas the proportion of cognitive items was almost doubled and at the same time behavioral and somatic items were completely removed as they did not perform well within the single-factor unidimensional depression scale. Our findings suggest that conceptual limitations may have resulted in favor of statistical fit.

The recommended next step within the RMT paradigm involves evidence-based conceptual review of the item-bank’s content to sufficiently and comprehensively define the concept of interest under measurement for the patient population for whom it will be used. RMT analysis provides statistical evidence for the scale’s measurement properties; however, this does not guarantee a scale’s content validity, as statistical cohesiveness in measuring a construct does not specify what the construct is or how it is conceptualized by the population for whom it is intended to be used with [1, 4, 6, 32, 33]. Therefore, statistical tests of a PRO instrument’s validity can mislead if the intended construct is not targeted sufficiently. Therefore, a conceptually driven empirical assessment of content validity, driven the population for whom the PRO is to be administered, would improve the ability of PROMIS depression to quantify the construct under measurement in this study sample.

In addition, outside any psychometric paradigm, but in line with clinical outcome assessment development guidelines, our findings further indicate that PROMIS depression psychometric analysis could benefit by refining of the context of use [4]. RMT analysis showed that the range of PROMIS depression items matched the range of depression reported by persons at the higher end of the continuum well. These findings could therefore be interpreted as supportive of the use of the item bank in populations with higher levels of depression. Contrary to this finding, item calibration for the PROMIS depression item banks was completed on a sample the vast majority of which (92%) was recruited from general not clinical population [17]. PROMIS depression psychometric analysis would therefore benefit by empirical exploration of the concept of interest in a predefined and specific context of use [4].

## Conclusions

In this study, RMT analysis supported the statistical scale performance of the PROMIS depression scales, but also identified targeting and sample measurement limitations. In practice, diagnosed anomalies may be resolved by attempting to explore the data and interpret the disparity between expected and observed scores of an evaluated scale further [3]. Within the RMT paradigm, rating scales and constructs are modified and, if necessary, more data are collected. In this respect, the application of RMT to the PROMIS depression item bank suggests that measurement could benefit by further examination of the construct under measurement and specifically the concept of interest and context of use.

## Data Availability

The datasets supporting the conclusions of this article are available from the PROMIS Health Organization, http://www.healthmeasures.net/explore-measurement-systems/promis

## Abbreviations

CTT: Classical test theory
IRT: Item response theory
PROMIS: Patient-reported outcomes measurement information system
RMT: Rasch measurement theory
